# The accuracy of the frontal extent in stereoscopic environments: A comparison of direct selection and virtual cursor techniques

**DOI:** 10.1371/journal.pone.0222751

**Published:** 2019-09-23

**Authors:** Chiuhsiang Joe Lin, Dino Caesaron, Bereket Haile Woldegiorgis

**Affiliations:** 1 Department of Industrial Management, School of Management, National Taiwan University of Science and Technology, Taipei City, Taiwan (R.O.C.); 2 Department of Industrial Engineering, School of Industrial Engineering, Telkom University, Bandung, Indonesia; National University of Singapore, SINGAPORE

## Abstract

This experiment investigated the accuracy of distance judgment and perception of the frontal extent in a stereoscopic environment. Eight virtual targets were projected in a circular arrangement with two center-to-center target distances (18 cm and 36 cm) and three target sizes (0.6 cm, 1.5 cm, and 3.7 cm). Fourteen participants judged the positions of virtual targets presented at a distance of 90 cm from them by employing two different interaction techniques: the direct selection technique and the virtual cursor technique. The results showed overall higher accuracy with the virtual cursor technique than with the direct selection technique. It was also found that the target size significantly affected the frontal extent accuracy. In addition, significant interactions between technique and center-to-center target distance were observed. The direct selection technique was more accurate at the 18 cm center-to-center target distance along the horizontal (x) and vertical (y) axes, while the virtual cursor technique was more accurate for the 36 cm center-to-center target distance along the y axis. During the direct selection, estimations tended to converge to the center of the virtual space; however, this convergence was not observed in the virtual cursor condition. The accuracy of pointing estimations suffered on the left side of participants. These findings could provide direction for virtual reality developers in selecting proper interaction techniques and appropriately positioning virtual targets in stereoscopic environments.

## Introduction

In recent years, the development of virtual reality (VR) has added a new dimension to human-computer interactions. In many applications of VR, a user interacts with virtual objects in a virtual environment (VE), such as for training in maintenance operations [1], manual assembly operations [2], and surgical simulations [3]. These applications mostly require observers to accurately interact with virtual objects at a particular distance. Among the various factors, interaction performance is evaluated on how accurately observers can judge the positions of the virtual objects in the VE. The observer can make distance estimates with either exocentric or egocentric distance references. Exocentric distance is the distance between two objects or points, and egocentric distance is the distance between the observer and the object. By using a metric of relative distance from the observer to the object, Cutting [4] classified space into three types: personal space, where the distance is within arm’s reach; the action space, where the distance is about 1.5–30 m; and the vista space, which is beyond approximately 30 m.

Spatial information such as distance, size, space, and relations are substantially important in the real world and VEs. Many recent studies have considered distance estimations and compared the accuracy of the estimates in the real world and VEs as their foundation of study [5, 6]. In addition, most of them have reported the accuracy of distance perceptions in in-depth planes, but only a few studies have been conducted in a frontal extent [7, 8]. Generally, the perception of distance may vary; it can be accurate, underestimated, or overestimated. Whenever the distances are systematically reported to be over- or underestimated, the overall space within all three dimensions may be perceived as compressed or expanded. In many of the studies on depth perception, underestimation of depth in VE is often reported. The insufficiency of the rendered VE [9], differences of judgment methods [10], and different viewing conditions [11] have been shown to contribute to distance compression. A holistic review by Renner, Velichkovsky [12] summarized the probable factors causing underestimation: technical factors, measurement methods, compositional factors, and human factors. Nevertheless, it is not completely understood why spaces are perceived as smaller in VEs, in contrast to the accurate perception of space in the real world.

One of the key issues in the perception of three-dimensional space is depth cues (i.e., sources of information about the spatial relations of the objects within the environment). A number of studies have shown the effects of depth cues on the perception of egocentric and exocentric distances (further review and analysis are available in [12, 13]). Investigations of the effects of environmental contexts on distance perception [14, 15] have reported that a continuous and homogeneously-textured ground surface was helpful for veridical distance perception. Another good example is research on the so-called action-specific effects on perception [16]. The results of that research indicated that the perception of various environmental and object properties, such as distance and object size, varies as a function of observers’ ability and intent to act in the environment. For example, observers wearing a heavy backpack might perceive an object as farther away because they would have to expend greater energy to walk to the object. Generally, depth or distance cues are classified as monocular or binocular [17]. Some cues are monocular; optic inputs in one eye are sufficient to extract distance information (e.g., occlusion, relative size, and relative density). Other cues are binocular; information from both eyes is combined to perceive the distance (e.g., binocular disparity, convergence, and accommodation).

The positioning of objects in the frontal extent is also a vital focus of study because of its importance for many applications in VEs [18]. Moreover, compared to studies in depth planes, studies on the distance estimation of objects in the frontal extent show a large variety of results. Geuss, Stefanucci [19] reported accurate judgments of approximately 100% of inter-object distances in VE. However, a more recent study by Kelly, Hammel [20] found under-perception of distance in the frontal extent in a grass-scene VE condition, but confirmed accurate estimation in the room VE condition. Another study, focusing on inter-objects in VE, also observed overestimation [21]. Considering the relationship between objects in the frontal extent of VEs, it is an important issue for various applications, and related information should be provided accurately [22]. Therefore, the present study focused on evaluating the accuracy of exocentric distances and the resulting perception of space in the frontal extent of a VE.

In general, two types of methods are employed to judge distance in VEs: action-based and non-action-based judgment. Action-based judgment involves walking, pointing, reaching, or combinations of these tasks and requires an observer to view a target and then perform the action. This judgment method is perceived to have greater validity because the actions are usually related to actions performed in both the real world and VEs, such as walking through or interacting within spaces [23]. A verbal report, an example of a non-action-based judgment, requires observers to view a target and then verbally report the perceived distance. The verbal report can also be described as conscious report/magnitude estimation of the distance to a target. It is well accepted that a verbal report is a simple way of measuring perceived distance; however, this type of report in the real word tends to be more variable and less accurate than action-based judgment [24].

Most previous studies have tried to estimate distance perception in VEs by using reporting methods such as perceptual matching techniques [25–28], verbal judgments [29, 30], blind walking [31, 32], triangulated blind walking [33], and timed imagined walking [34]. Recently, VR applications have become more interactive, allowing a user not only to visualize a 3D image but also to interact with the 3D objects in the VEs. For such interactions, researchers have investigated a 3D mid-air input technique [35–37] as an alternative to conventional 2D input devices (e.g., trackball, mouse, etc.) and touch screens [38]. Generally, the technique employs a 3D target and pointing hand movement as an input function for various systems requiring freehand or touchless computer interactions. The challenge is to determine whether such direct-hand pointing interactions have comparable performance to those of traditional 2D input devices.

A recent work by Lin and Woldegiorgis [39] investigated the performance of direct-pointing wherein participants directly moved their hands to reach for real/virtual targets projected in front of (negative parallax) the VE projection display. The results revealed that participants tended to overestimate the depth by approximately 10 cm, and that the overestimation decreased as the depth increased. Moreover, the direct-pointing method was claimed to reduce the underestimation problem that is commonly reported in VEs. A similar method (direct-pointing) was employed to estimate the distance of virtual targets presented in the frontal extent at three depth levels [40]. The study concluded that compression occurred in the frontal plane and that observers tended to underestimate the depth at 100 cm and 150 cm. Bruder, Steinicke [41] and Swan, Singh [28] employed direct-reaching with the hand as a reporting method to reach virtual targets. In Bruder, Steinicke [41], a comparison of interaction techniques between 3D mid-air and 2D touch screen controls indicated no significant differences in the error rates of target selection in a stereoscopic environment. In Swan, Singh [28], a comparative study between direct-matching and blind reaching in estimating the positions of mixed real and virtual targets in a stereoscopic environment indicated that direct-matching was more accurate than blind reaching. In our study, these techniques (direct-pointing, matching, and direct-reaching) are considered direct interaction techniques.

In contrast to a direct interaction technique, which requires direct involvement with the object rather than communicating with an intermediary [42], an indirect interaction technique allows a user to use a physical control (e.g., sliders, joysticks, etc.) to control an icon (e.g., a virtual cursor, a virtual hand cursor) to perform a specific task [43]. Previous studies have applied the indirect interaction technique in VEs. Bruder, Steinicke [44] compared three different approaches for selecting virtual targets: direct input, distant input with a virtual offset cursor (white marker), and distant input with a virtual hand cursor (hand cursor). In the direct input approach, participants positioned the tips of their index fingers on the target, and in the virtual offset and hand cursor conditions, they moved a white marker or hand cursor to the virtual target. The results revealed that subjects made fewer errors in selecting a virtual target at different heights above a 3D tabletop setup when using an input with an offset cursor than when they used direct input and the offset hand. Poupyrev and Ichikawa [45] compared an interaction metaphor of a virtual pointer (the ray casting technique) and a virtual cursor (direct input with a virtual offset cursor) for object selection and repositioning tasks. The comparison showed that the virtual pointer (considered an indirect interaction technique) exhibited more accuracy in the selection of objects within reaching distance than did the virtual cursor (considered a direct interaction technique). A recent study by Deng, Geng [46] asked participants to position a ball-shaped object in a spherical area in a virtual space using head tracking or handheld controllers. These techniques allowed a virtual light ray emitted from the controllers to move the object. Studies on interaction techniques (direct- and indirect interactions) and the extent to which this factor influences the accuracy of distance estimation are summarized in the following section (see Table 1 for an overview of studies on interaction techniques).

**Table 1:** Overview of studies on interaction techniques. The interaction technique affects the accuracy of distance estimation/target selection in different spatial perspectives and displays under various experimental conditions.

Author/s (number in reference list)	Display^a^	Spatial Perspective (Egocentric/Exocentric Distance)	Interaction Technique	Experimental Conditions	Results (Findings)^b^
Bruder, Steinicke [44]	Tabletop setup	Exocentric distance	Direct mid-air selection with the tip of the user’s index finger	Direct input with user’s fingertip vs. offset based input with a virtual offset cursor	Offset-based input was less accurate than direct input with the user’s fingertip.
Bruder, Steinicke [41]	Tabletop setup	Exocentric distance	Direct mid-air selection with the tip of the user’s index finger and 2D touch screen	3D mid-air selection vs. 2D touch screen	Not significant
Lin, Abreham [47]	Projection screen and HMD	Egocentric distance	Direct mid-air selection using a pointing stick	Stereoscopic vs. immersive environments	The immersive environment was less accurate than the stereoscopic environment. Overestimation was found in distance estimates.
Lin and Woldegiorgis [39]	Projection screen	Egocentric distance	Direct mid-air selection using a pointing stick	Stereoscopic vs. real environments Pointing by vision vs. memory	The stereoscopic environment was less accurate than real world. Not significant Overestimation was found in distance estimates.
Lubos, Bruder [37]	HMD	Exocentric distance	Direct mid-air selection with the tip of the user’s index finger	Selection along a view direction vs. selection along a movement direction	Selection along view direction was less accurate than along movement direction.
Napieralski, Altenhoff [10]	HMD and real-world	Egocentric distance	Direct reaching using a stylus and verbal response	Direct reaching vs. verbal responses Immersive virtual environment vs. real world	Significant difference between interaction techniques Underestimation was found in distance estimates.
Poupyrev, Weghorst [48]	Desktop monitor	Egocentric distance	Indirect-input with 6DOF controller	Absolute mapping control vs. relative mapping control	Not significant
Werkhoven and Groen [49]	HMD	Egocentric distance	Direct-input with virtual hand control and indirect-input with 3D cursor control	Virtual hand control vs. 3D cursor control Monoscopic virtual environment vs. stereoscopic virtual environment	3D cursor control was less accurate in positioning task than virtual hand control. The stereoscopic condition was more accurate than the monoscopic condition.
Woldegiorgis and Lin [40]	Projection screen	Exocentric distance (frontal plane)	Direct mid-air selection using a pointing stick	Stereoscopic vs. real environments	Accuracy was higher in the real environment than in the stereoscopic environment. Space compression was found in distance estimates.

^a^HMD, head-mounted display.

^b^Not significant indicates a not significant difference in accuracy under the described experimental conditions.

Although it is as important as perception, interaction performance (distance accuracy) and especially the effects of the interaction techniques in VEs have not yet been studied as much as visual perception. Therefore, the present study investigated the effects of interaction techniques on the accuracy of distance estimation in a projection stereoscopic display. Moreover, even though some previous studies (Table 1) have analyzed the performance of interaction techniques in VE, they considered the techniques separately, focusing on either the direct or the indirect interaction technique. This study therefore attempted to compare direct interaction and indirect interaction techniques in terms of their effects on the accuracy of distance estimation. To provide comprehensive knowledge of the spatial information, distance estimation in the frontal plane needs to be evaluated.

In the present study, the accuracy of distance estimation in the frontal plane of a stereoscopic environment using two interaction techniques (direct-selection and virtual cursor) was studied. We evaluated these two interaction techniques in selecting a 3D stereoscopic object in front of the projection screen display. We used a standard selection task (ISO 9241–9) to determine differences in 3D object selection performance for varied sizes of the targets and varied center-to-center distances between targets. The results of this study should provide direction for the choice of interaction techniques, as well as the optimal sizes and distances between targets in stereoscopic environments.

Based on the results of previous related studies [6, 8, 40, 41, 44, 49], the present study tested the following hypotheses:

H1: Interaction technique affects the accuracy of distance estimation in the VE, with the virtual cursor technique being more accurate than the direct-selection technique.

H2: Space is compressed (underestimated) in the frontal extent of the VE.

H3: The center-to-center distance accuracy is higher for both narrower inter-object distances and larger target sizes.

## Methods

The purpose of the experiment was to investigate the accuracy of distance estimation and perception of the frontal extent in a stereoscopic environment, where two interaction techniques, namely, the direct-selection and virtual cursor techniques, were considered. The two interactions of the direct-selection and virtual cursor techniques were developed based on interaction terms between users and targets in VE by Mine [43].

### Direct selection of virtual objects

3D interaction in VEs has been the focus of many research groups over the last few decades [50]. Direct interaction provides the most natural type of interaction with virtual objects [37]; however, this technique leads to confusion because the participant is touching an intangible object, or touching a void [51]. In addition to distance estimation being less accurate in VE than in the real world, direct interaction can also lead to double vision and vergence-accommodation conflicts [52]. Although this technique introduces visual conflicts, most results from similar studies agree that direct interaction could significantly improve the performance of object manipulation [53] because optimal performance may be achieved when visual and motor spaces are coupled closely [54, 55].

**Fig 1:**
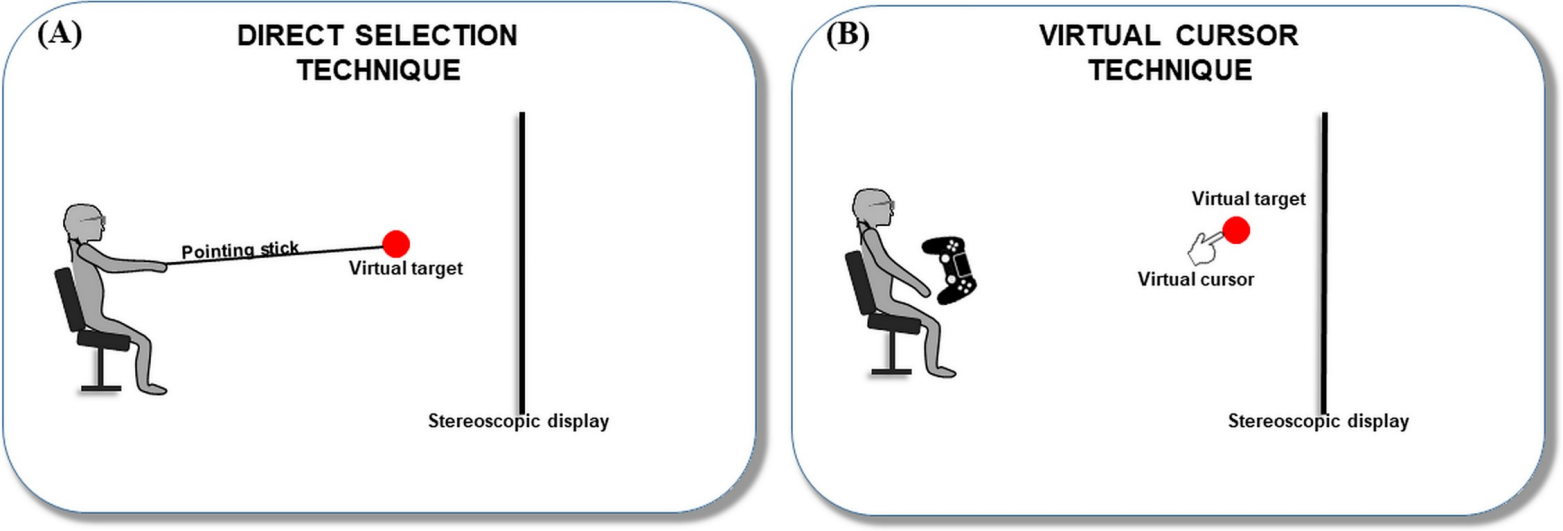
Illustration of the two interaction techniques. (A) The direct selection technique involves direct reaching with a pointing stick; (B) the virtual cursor technique involves a virtual cursor controlled with a gamepad.

Direct interaction is an impression of direct involvement with an object, rather than of communication with an intermediary [42]. In our study, in the direct interaction condition, participants estimated the distance by pointing to the outer surface of a spherical virtual target (Fig 1A). This reporting method is similar to one of the two methods employed in Napieralski, Altenhoff [10], wherein a physical arm was employed to estimate a target depth in VE. Since the distances considered in their experiment were within arm’s reach, pointing by hand was sufficient. However, in the present study, we used a physical object (i.e., a pointing stick) with a marker attached to the tip to be detected by the tracking system.

### Indirect selection of virtual objects

Indirect interaction requires conversion between input and output [42], such as a slider on a panel that controls the intensity of light; in this case, a user directly controls the intermediary slider and indirectly controls the light. In our study, for indirect interaction, participants estimated the distance by moving a virtual cursor controlled with a gamepad (Fig 1B). They needed to place the virtual cursor (represented by a hand cursor) at the center of the surface of the virtual target. In this type of interaction, both the virtual cursor and the virtual targets were displayed stereoscopically, eliminating the mismatch between the real and virtual objects. This approach may reduce the visual conflicts which commonly occur when a user is trying to select a virtual object with a physical object (e.g., the user’s real finger). However, it is not clear whether the reduction of visual conflicts can improve overall selection task performance.

### Participants

Fourteen participants, eleven males and three females aged between 23 and 31 years old (M = 24.64, SD = 2.06), were recruited. All participants self-reported normal or corrected-to-normal vision and right-hand dominance. Regarding experience with electronic systems, most of the participants were familiar with the input devices. Three of them had never held a gamepad, and the rest had operated gamepads before. None rated themselves as experts in their use. Among all the participants, only three had previous experience in virtual reality. Prior to the experiment, all participants had to pass a stereo vision test by viewing a virtual target projected 90 cm from them. All participants who qualified were invited to participate in the experiment. Written informed consent was provided to each participant prior to the experiment. The participants were given explanations and were aware of the aims of the experiment, and they volunteered to experience a virtual environment. The participants received neither any form of payment nor compensation with academic credit. The experiment was approved by the research ethics committee of National Taiwan University (NTU-REC No: 201209HS002).

### Experimental variables and design

The experiment considered two interaction techniques, two center-to-center (c2c) distances between targets, and three target sizes. Therefore, a combination of three independent variables, namely, interaction technique (direct pointing and distant cursor), c2c distance (18 cm and 36 cm), and target size (0.6 cm, 1.5 cm, and 3.7 cm), were used and tested on all the participants (within-subject design). After being divided into two equal groups, the participants were assigned to start with one of the two techniques (direct pointing or distant cursor). Once the technique was chosen, the c2c distance and target size were varied randomly. In both the direct selection and virtual cursor conditions, the participants were presented with two levels of c2c target distance and three levels of target size. After completing all the trials in the first round of the experiment, the participants returned at least two days later to complete the other condition. The dependent variables were the accuracies of the x-position, y-position, and exocentric distance. Based on the results of the three accuracy measures, a perceived frontal extent in VE could be evaluated. In addition, the task completion time, defined as the time for the participant to complete the pointing task with eight targets, was measured.

The participants estimated the target positions by pointing to the center of the target surface with a pointing stick (direct selection technique) or by controlling the movement of a virtual cursor with a gamepad (virtual cursor technique). During the task, the data of the reflective marker (attached to the tip of the pointing stick) positions in the direct selection technique, tracked by six infrared cameras at a rate of 120 frames per second, were collected. In the virtual cursor condition, the positions of the virtual cursor and reference targets within the VE were recorded by a Unity 3D system. The data collected were then organized to analyze how close the estimates were to the corresponding reference targets. First, a positional estimate of a single object along the x- and y-axes were collected, and then the accuracies of the x- and y-positions were further evaluated. Second, the position data of the c2c distance between two consecutive (with respect to pointing order) targets were then used to calculate the accuracy of exocentric distance. After that, the overall positional perception of the targets was used to evaluate whether space was compressed (underestimated) or expanded (overestimated) in the frontal plane. The accuracy was calculated using the following formula [39, 56],

$$Accuracy=\left(1-\left|\frac{De-Da}{Da}\right|\right)$$ (1)

where *De* indicates the participant’s estimated position/distance, obtained from the data recorded under the two techniques, and *Da* is the corresponding actual or reference position/distance. A value closer to one indicates the estimation was more accurate.

### Experimental setup and stimuli

**Fig 2:**
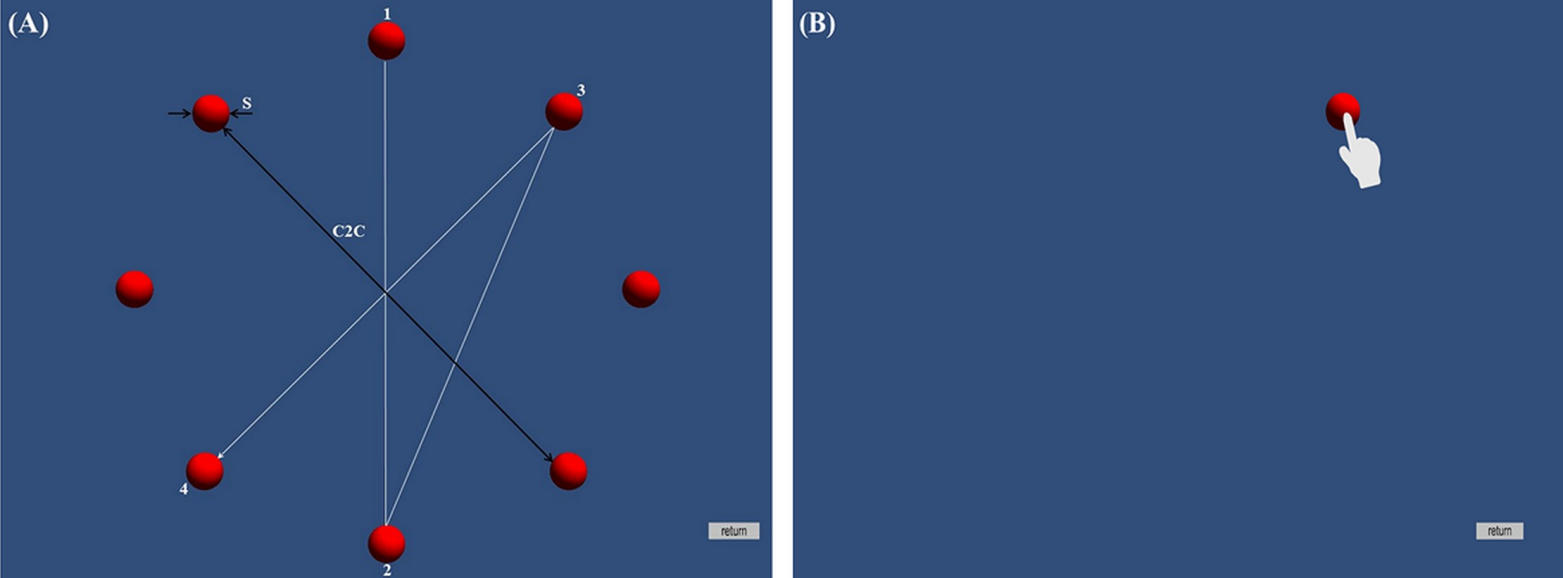
The experimental scenario. (A) Eight virtual targets were displayed in a circular arrangement, and their sizes (s) and center-to-center (c2c) distances between targets were altered. The targets appeared one at a time in sequence until all targets were displayed. (B) The virtual cursor in the virtual cursor condition was controlled with a gamepad to acquire the target.

**Fig 3:**
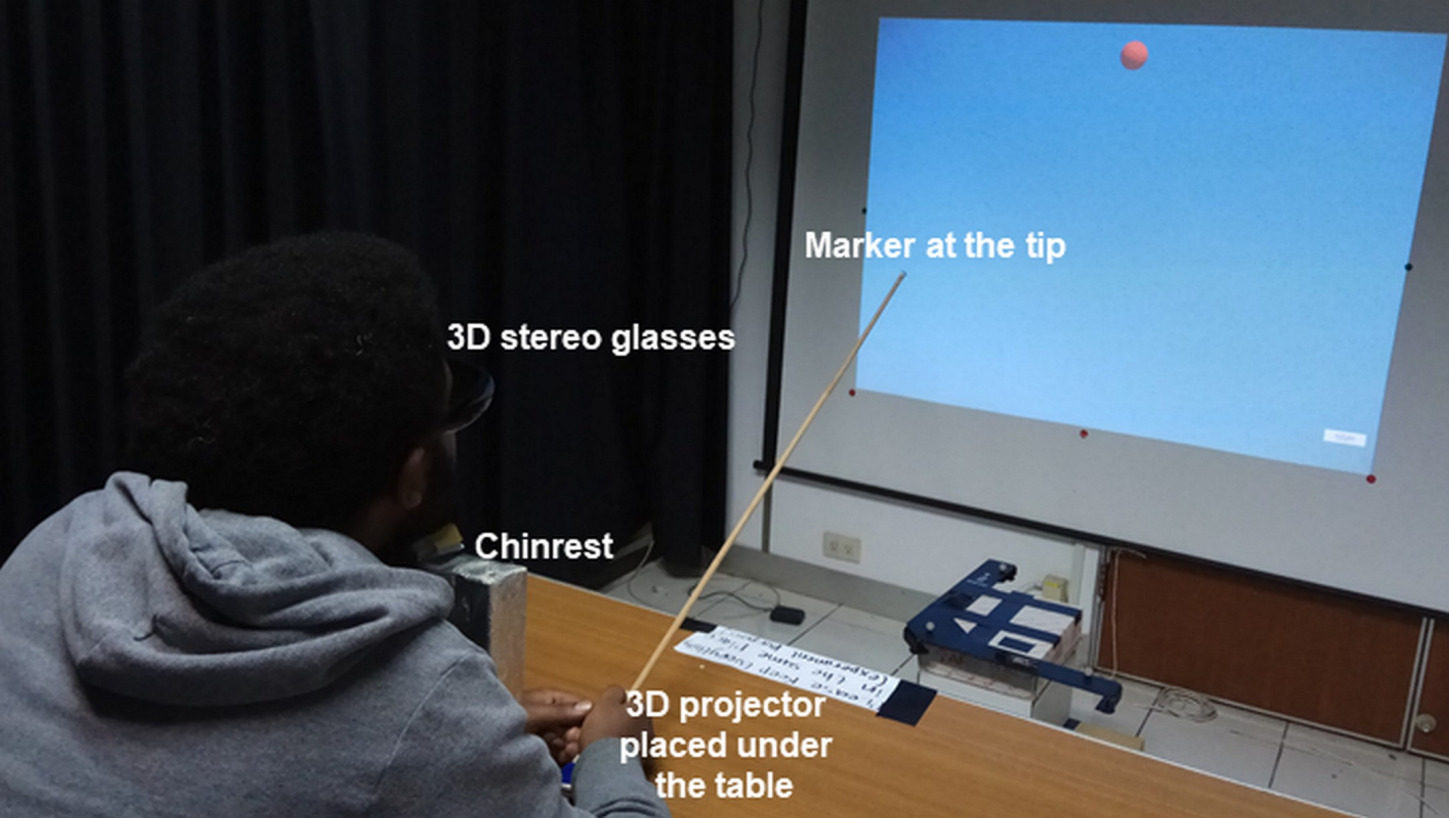
The experimental setup. The participants estimated the positions of the virtual targets by direct selection technique. The participants wore 3D glasses, and their chins were fixed at a distance of 210 cm from the projection screen. The individual in this manuscript has given written informed consent (as outlined in the PLOS consent form) to publish these case details.

The experimental space was 4.6 m x 3.2 m x 2.5 m in size and partitioned by black curtains to create an excellent stereoscopic environment. The VE and stereoscopic targets were developed in Unity 3D. The targets were displayed in the order specified by ISO [57]. The eight targets appeared one at a time in sequence until all were displayed (Fig 2A). The VE was projected by a ViewSonic 3D projector onto a projection screen that was 130 cm wide x 100 cm high. The VE space was a uniform dark blue (Fig 2). The participants wore NVIDIA 3D glasses integrated with a 3D emitter to perceive stereoscopic vision. The stereoscopic targets were displayed at a distance of 90 cm from the participant. The participant and the projector were placed at a fixed position of 210 cm from the projection screen (as shown in Fig 3). The origins for reference measurement in both the direct selection and the virtual cursor conditions were set at the center of the projector, which was placed under the table (75 cm above the floor) and perpendicular to the participant’s eyes.

The pointing task in the direct selection technique was performed using a light wooden stick of length 80 cm. The material was carefully selected so that the weight of the stick would not affect participants’ pointing posture or performance. A 0.6 cm reflective marker was attached to the tip of the stick. A wireless remote control for confirming pointing judgment was also attached to the lower end of the stick. In the virtual cursor technique, a dual analog gamepad was used to control a virtual cursor the along x-, y-, and z-axes within the VE. The gamepad’s left analog stick controlled a virtual cursor in two-degrees-of-freedom (DoF) translation (up and down, left and right), including diagonal movements. The gamepad’s right analog stick was used to control the depth (forward and backward) of the virtual cursor. The size of the virtual hand cursor was scaled approximately to the size of each target to provide good visualization when touching the target. In both the direct selection and virtual cursor conditions, the initial positions of the tip and virtual cursor were kept fixed. Using the center of the projector as the reference of measurement, the relative positions of the initial points of the tip and the virtual cursor were about 82 cm diagonally to the left with respect to the origin. The position of the cursor was reset to the initial point for each trial. Applying more force on the gamepad resulted in higher speed. Prior to conducting the experiment, a preliminary experiment was conducted to obtain the optimum sensitivity value of the gamepad. The sensitivity was set to the value that enabled the user to control the virtual cursor for precise and fast movement. We optimized the gamepad control with respect to the gain between exerted force and virtual cursor speed. The resulting average sensitivity value was set to approximately 2 m/s. Participants pressed the “X” button on the gamepad grip to confirm the pointing judgment. For both conditions, no visual feedback (e.g., change in color or shape) was given when the target was chosen, other than the appearance of the next target.

### Procedure

The participants completed all the trials of the experiment in two sessions, one for the direct selection technique and the other for the virtual cursor technique, separated by at least two days to minimize the effects of learning and fatigue. Prior to the experiment, the participants completed a consent form detailing the purposes and procedures of the task. An equivalent verbal explanation was also given while their interpupillary distances (IPDs) were measured.

A fixed chinrest was placed on the tabletop for the participants to rest their chins to minimize the possibility of differences in viewing perception due to head movements. The participants were asked to familiarize themselves with the experimental setup; i.e., they were instructed to view the VE and to obtain a clear image of the virtual targets. The experimenter introduced the procedure and showed them how to employ the interaction technique to be used in the first session. Then the participants practiced two or three trials until they completed a trial without procedural errors. The first experimental session for the selected technique followed the practice session. Each participant completed a total of 96 trials (2 techniques x 3 target sizes x 2 c2c distances x 8 targets) in two sessions of about 30 minutes each.

## Results

Repeated-measures ANOVA was used to evaluate the accuracies of the x-position, y-position, and exocentric distance for the three independent variables. Corresponding to the accuracies of the x-position, y-position, and exocentric distance, the perception of virtual space in the frontal extent could also be evaluated.

### Accuracy of x-position, y- position and exocentric distance

**Fig 4:**
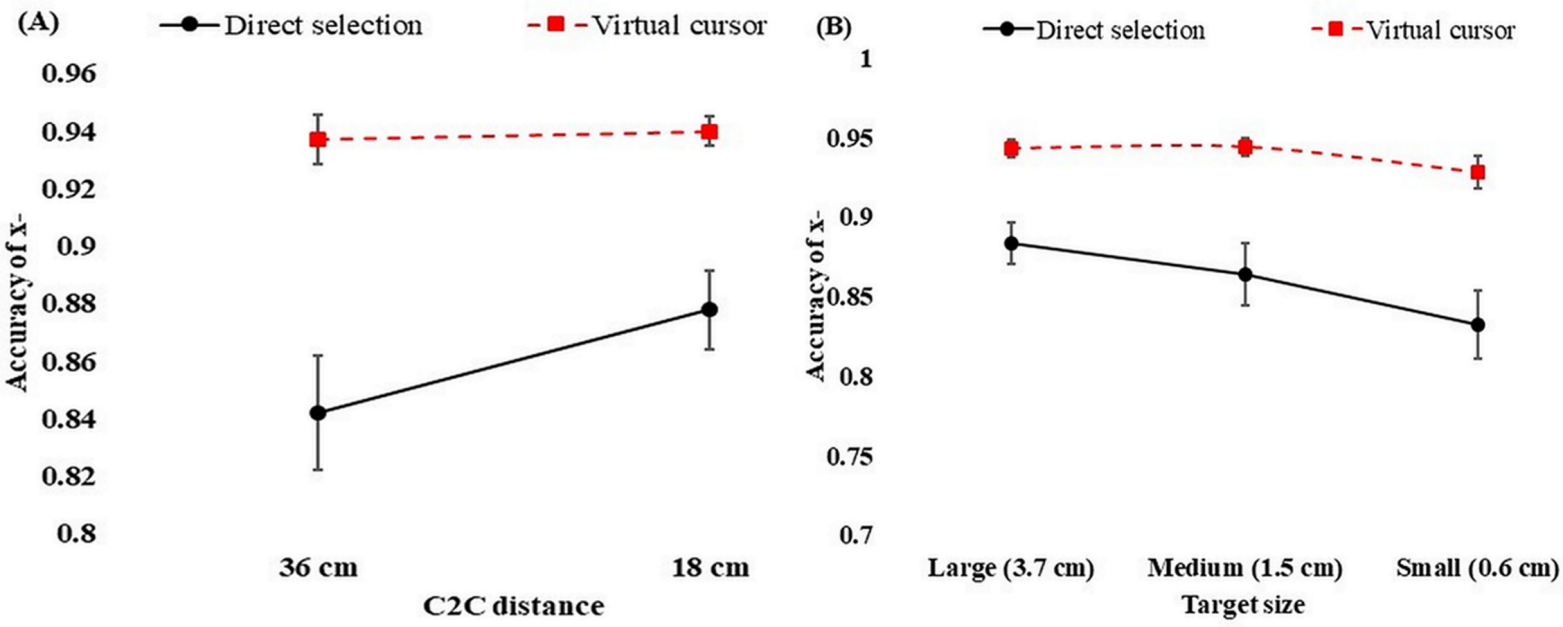
Accuracy of x-position in the c2c distance (A) and interactions of target size and technique (B). The error bars represent standard error of the mean.

The accuracy of the x-position was significantly higher in the virtual cursor condition (M = 0.94, SD = 0.01) than in the direct selection condition (M = 0.86, SD = 0.02). The results of the ANOVA indicated a significant difference in accuracy between the two techniques (F [1, 13] = 21.60, *p* < 0.001). The results also revealed significant interactions between technique and c2c distance (F [1, 13] = 5.80, *p* = 0.032) in the x-position. Fig 4A illustrates the interaction between c2c distance and technique. It appears that the participants were able to identify objects more accurately at the 18 cm c2c distance than at the 36 cm c2c distance with the direct selection technique. On the other hand, no differences of accuracy were found for the virtual cursor technique for selecting objects displayed at 36 cm (M = 0.937, SD = 0.01) and 18 cm (M = 0.940, SD = 0.006) of c2c distance.

The results of the ANOVA revealed that target size significantly affected the accuracy of the x-position (F [1.96, 25.43] = 4.88, *p* = 0.017). The accuracy was marginally better for a target size of 3.7 cm (M = 0.91, SD = 0.01) than for the sizes of 1.5 cm (M = 0.90, SD = 0.01) and 0.6 cm (M = 0.88, SD = 0.01). However, there was no significant difference in accuracy between the c2c distances for the x-position (F [1, 13] = 2.44, *p* = 0.142).

The interaction technique also influenced the estimation of the y-position (F [1, 13] = 30.04, *p* < 0.001). Participants estimated the target more accurately with the virtual cursor technique (M = 0.93, SD = 0.11) than with the direct selection technique (M = 0.80, SD = 0.03). Furthermore, the accuracy of the y-position was also affected by target size (F [1.64, 21.28] = 5.38, *p* = 0.017). Tukey’s test showed that the accuracy was significantly lower for the 0.6 cm target size (M = 0.84, SD = 0.01) than for the 1.5 cm (Mean = 0.88, SD = 0.01) and 3.7 cm target sizes (Mean = 0.89, SD = 0.01). However, the main effect of the c2c distance (F [1, 13] = 0.34, *p* = 0.573) on the accuracy of the y-position was not significant.

**Fig 5:**
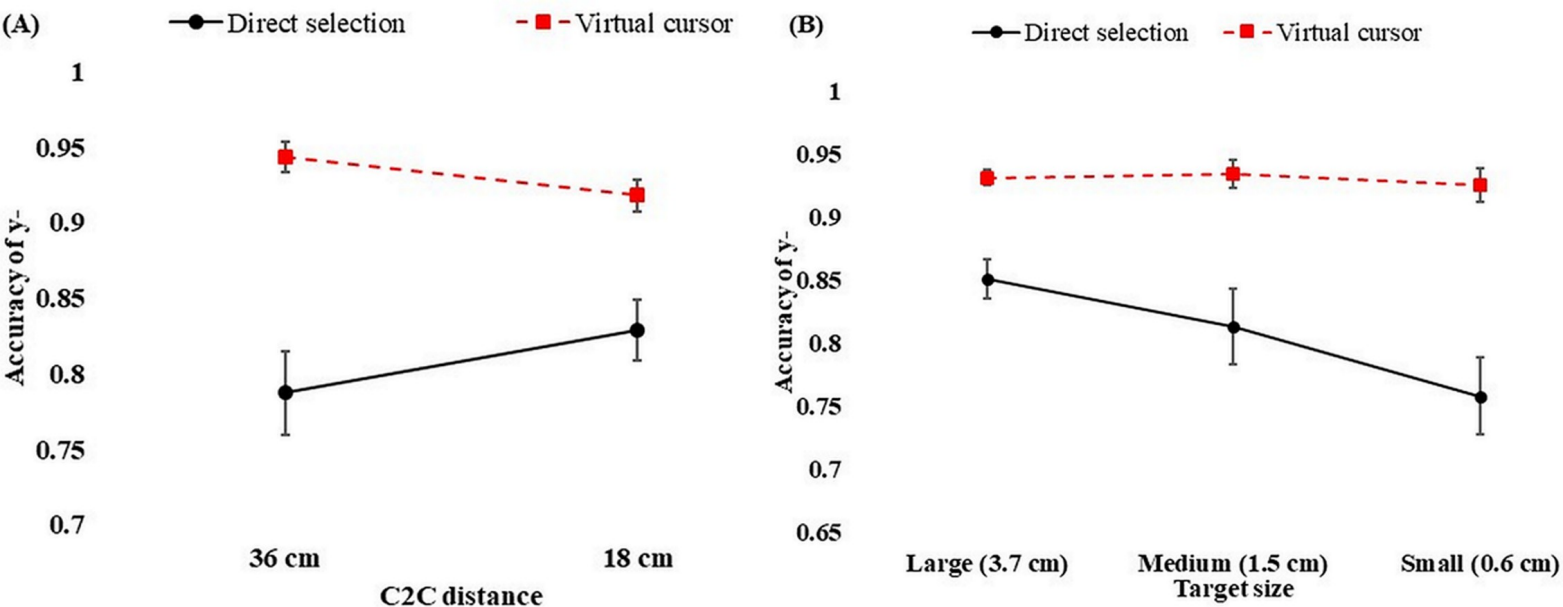
Accuracy of y-position in c2c distance (A), and interaction of target size and technique (B). The error bars represent standard error of the mean.

The interactions between c2c distance and technique (F [1, 13] = 16.94, *p* = 0.001) and target size and technique (F [1.52, 19.78] = 5.07, *p* = 0.024) had significant effects on the accuracy of the y-position. As shown in Fig 5A, the direct selection technique was more accurate at the 18 cm distance than at the 36 cm distance between targets. For the virtual cursor technique, the opposite trend was found: Judgments were more accurate for the c2c distance of 36 cm than for that of 18 cm. From this study, it is evident that, with respect to the accuracy of the x- and y-positions, the direct selection technique was more accurate for the narrower inter-object distance, while the virtual cursor technique was more accurate for the wider inter-object distance in the y- (vertical axis) position. However, for the x- (horizontal axis) position, there were no significant differences in accuracy for the 36 cm and 18 cm inter-object distances when the virtual cursor technique was used. Regarding target sizes, accuracy was marginally higher for the largest target size (3.7 cm) with both techniques than for the other two sizes.

The results of exocentric distance measurement showed that accuracy was significantly higher (F [1, 13] = 37.70, *p* < 0.001) in the virtual cursor condition (M = 0.95, SD = 0.01) than in the direct selection condition (M = 0.88, SD = 0.01). A significant difference in the accuracy of exocentric distance was also found for target size (F [1.79, 23.27] = 3.85, *p* = 0.04). The corresponding mean accuracies were 0.92 (SD = 0.01), 0.91 (SD = 0.09), and 0.90 (SD = 0.01) for the target sizes of 3.7 cm, 1.5 cm, and 0.6 cm, respectively. We found no significant differences for the varied c2c distances on accuracy of exocentric distance (F [1, 13] = 1.63, *p* = 0.224).

### Perception of virtual space in the frontal extent

**Fig 6:**
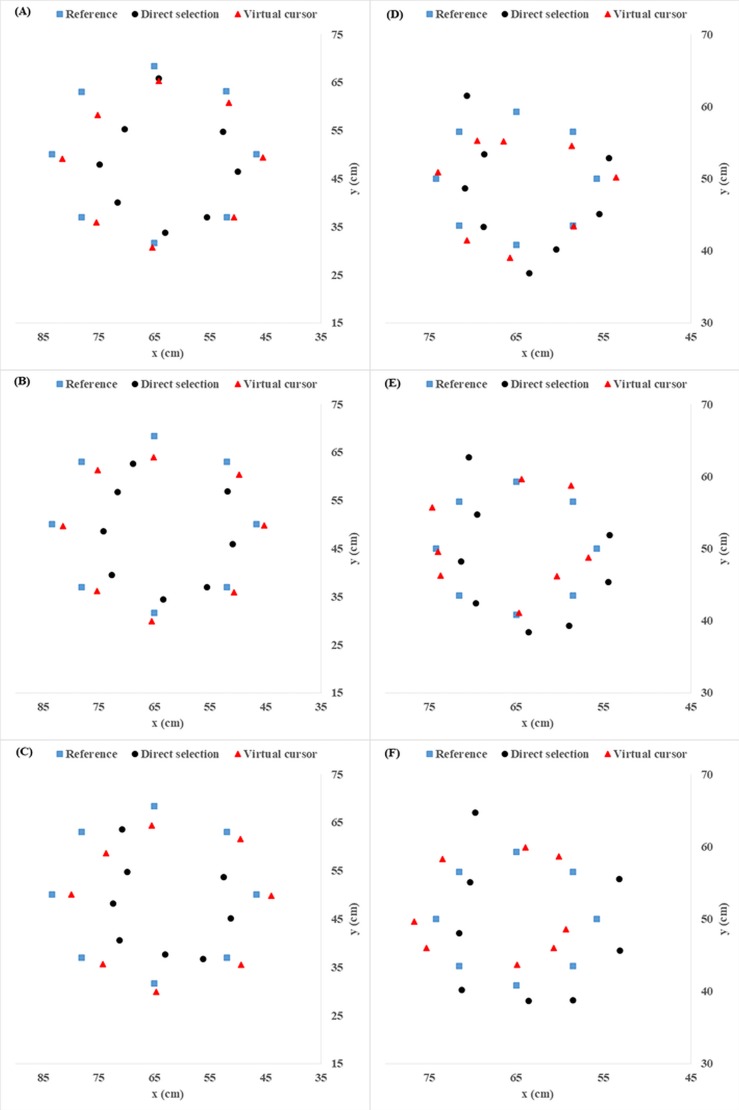
The overall mean of pointing estimations with respect to reference targets in direct selection and virtual cursor conditions at 36 cm of c2c distance for (A) 3.7 cm target size, (B) 1.5 cm target size, and (C) 0.6 cm target size. The figure also compares the overall mean of estimates of pointing with respect to a reference target at 18 cm of c2c distance, for (D) 3.7 cm target size, (E) 1.5 cm target size, and (F) 0.6 cm target size.

The pointing estimations with the two interaction techniques with respect to targets with c2c distances of 36 cm and 18 cm are shown in Fig 6. It can be observed that when direct selection was employed, then in the x- and y-positions of the wider c2c distance (36 cm), underestimation occurred because the points were concentrated close to the center (Fig 6A–Fig 6C). The figures also show underestimation of the exocentric distance in the direct selection condition. The overall direct selection estimations for the narrower c2c distance, 18 cm, were systematically shifted slightly to the right with respect to the reference targets (Fig 6D–Fig 6F). Therefore, from the plotting of pointing estimations, it was determined that, when the distance between objects was wider and the direct selection technique was employed, the frontal view distance estimations were compressed.

Considering the target sizes shown in Fig 6D–Fig 6F, the estimations of positions with both techniques for the smallest target size (0.6 cm) were more dispersed than those for the other two sizes (1.5 cm and 3.7 cm). Consequently, smaller target sizes and narrower c2c distances led to less accuracy with both techniques.

### Task completion time

Task completion time was measured from the moment the participants judged the first target until they completed the task and all eight targets had appeared. The c2c distance exhibited significant effects on completion time (F [1, 14] = 13.98, *p* < .005). The average completion time was M = 13.20 (SD = 0.93) for the narrower distance of 18 cm, while for the wider distance of 36 cm, it was M = 15.28 (SD = 1.34). However, the main effects of technique (F [1, 14] = 3.25, *p* = .095) and target size (F [1.46, 18.97] = 0.38, *p* >.05) were not statistically significant. The interaction of c2c distance and technique had a significant effect on completion time (F [1, 13] = 5.85, *p* < .05). The interaction of technique and target size also showed a significant effect on completion time (F [1.34, 17.40] = 7.90, *p* < .05). The pairwise contrast was significant (*p* < .005) for the smallest target size. The results revealed that the participants took significantly longer with a virtual cursor than with the direct selection technique when targets were displayed with the smallest target size (0.6 cm).

## Discussion

This study evaluated the accuracies along the x- and y-directions and also the exocentric distance of the frontal extent of the stereoscopic environment with two interaction techniques, two c2c target distance levels, and three target sizes. The positions of pointing estimations were then used to analyze the observers’ perceptions of the frontal extent.

### Accuracy of x-position, y-position and exocentric distance

The accuracy results for the x- and y-positions showed that the interaction technique played an important role. From the results, it is observed that the virtual cursor technique was more accurate than the direct pointing technique. This result was in line with the finding of a previous study [44], which revealed a smaller error rate for a virtual cursor than for direct pointing in target selection under a stereoscopic environment. Although different techniques and different 3D displays were employed in Bruder, Steinicke [44], one of the main findings was that the direct selection technique was less accurate than the virtual cursor technique. A plausible explanation for this difference is the visual conflicts or misperceptions apparent in the direct pointing technique, where the virtual target appears blurred, in contrast to the pointing stick, which appears sharp [58]. However, in the present study, the participants reported no visual conflict issues when the direct selection technique was employed.

In the virtual cursor condition, a virtual hand cursor was placed on the outer surface of the spherical virtual target. However, occlusion would occur if the virtual cursor passed through the virtual target, and the cursor always occluded the spherical virtual target; thus, the participant might realize that the cursor had passed behind the target. Although no visual feedback was given (e.g., color or shape change) when the cursor touched the target, the appearance of the hand cursor could serve as a visual cue that contributed to the higher accuracy results of the x- and y- positions as compared to the direct selection technique. Another visual cue that might have affected the accurate judgment of distance in the virtual cursor condition was the relative size cue. Since the size of the virtual hand cursor was scaled approximately to the size of each target, then as the hand cursor moved towards the target, the user could compare the sizes of the cursor and target until they were similar, which might have indicated that they were at the same depth. In other words, when the hand cursor was smaller than the target, the cursor was farther from the target. Thus, this relative size cue might have resulted in more accurate selection.

Contrary to expectations, the present study did not find a significant difference between narrower (18 cm) and wider (36 cm) c2c target distances in the accuracies of the x-position, y-position and exocentric distance. This result is inconsistent with those of previous studies, such as Lin, Woldegiorgis [6], which reported a marginally significant difference at one shortest distance of 10–20 cm between targets. This difference might be accounted for by the differences in the judgment methods and the experimental tasks. In the experiment of Lin, Woldegiorgis [6], perceptual matching by sketching the distance between two real and virtual targets was used. In contrast, the present experiment used a reciprocal tapping task with the more direct interaction of reaching/pointing (direct selection and virtual cursor) to the target referent, so the insignificant differences in the accuracies obtained in the present experiment could have been partly due to the consistency of accuracy obtained by the judgment methods. We also speculate that the two fixed c2c distances between targets used in this study contributed to the insignificant differences in accuracy. A future study with a greater focus on c2c target distance, a factor which may have contributed to the difference in distance accuracy, is therefore suggested.

Another factor that might have influenced the accuracy of the x-position, y-position, and exocentric distance was target size. We found that estimations for the largest target (3.7 cm) were marginally better than those for the medium (1.5 cm) and small (0.6 cm) targets. These results confirm that target size affected the estimation, with accuracy increasing with the target size [59]. Moreover, these findings provide further support to the idea that accurate distance estimation with respect to target size in the real world can also be generalized to VEs. The overall estimation accuracies of the x-position, y-position, and exocentric distance were approximately 93% for the distant cursor method and 85% for the direct selection technique.

This study is distinctive because it has combined x-position, y-position, exocentric distance, and interaction technique, unlike previous VR studies. Despite differences in the distance reporting techniques and measurement accuracy of the previous studies [39, 44, 59] and the present study, the results confirm that direct selection of virtual objects can be used as a potential response method [60, 61] in stereoscopic environments. In addition, the virtual cursor technique has the potential for high accuracy of virtual object estimation [44].

### User perception of the frontal extent

In this study, the perception of frontal extent was also analyzed based on the pointing estimations. From Fig 6, it can be observed that pointing estimations were concentrated at the center, particularly during the direct selection technique at the 36 cm c2c distance. This finding is in substantial agreement with recent studies [20, 40, 62] showing that the user’s frontal extent perception is smaller than the actual space in a stereoscopic environment. However, the compression in the frontal extent was not so visible for the virtual cursor technique. These differences may be explained by the fact that in this study, both the cursor and the targets were displayed stereoscopically, so the virtual space compression may have been reduced [44], which could have been induced by the variation of the references in the stereoscopic environment. Another possible reason is the familiar size of the objects and a higher sense of presence, particularly in the virtual cursor condition. If the size of an object (i.e., a hand cursor) is familiar to the observer, absolute distance information is available. A study by Interrante, Ries [63] added not only familiar objects to an unfamiliar environment but also used a virtual replica of a room that the participants had seen before as the virtual environment. The results revealed that the participants did not underestimate the virtual distances. In a follow-up study, the participants underestimated distances in both enlarged replica and shrunken replica conditions [64]. The authors concluded that the good estimates might have been due to a higher sense of presence. These findings are also supported by Sun, Li [65], who found that increased involvement of the users due to visual cues (i.e., avatars) can improve user performance in virtual environments. The described studies [63–65] have shown consistently that adding familiar objects to enhance a sense of presence can improve distance estimations. In the present study, although the virtual environment was presented in a sparse environment (i.e., spheres in front of a dark blue background) for both conditions, only the virtual cursor condition contained a familiar size cue (i.e., the hand cursor), and its appearance might have enhanced the sense of presence during the pointing to or selection of the target compared to the direct selection condition. Thus, these findings suggest that a low sense of presence in the direct selection condition might be a potential cause of underestimation. However, at this stage, it might be difficult to conclude that direct interaction by reaching or pointing to the target can cause underestimation of the frontal extent, for few comparative studies have applied this direct interaction technique. Further experimental investigations are needed to answer the question of whether the underestimation of the frontal extent is due to the low sense of presence in stereoscopic environments.

Nevertheless, the compression of space perception was not so apparent for the narrower c2c (18 cm) distance with the two techniques. Therefore, further experimentation with the user’s virtual space perception in the frontal extent for various ranges of c2c distance might be needed to fully understand the effect of the interaction of technique and c2c distance. In the present study, the effect of depth (z-axis) on x- and y-position accuracy was not specifically studied. Although the participants were instructed to select the target as accurately as possible within the same frontal plane, the pointing inaccuracy on the z-axis could have played a part in the variation of estimations. Further studies taking this variable into account will be needed to clarify the possible effect of depth on frontal plane estimations.

The estimation of positions with both techniques for the 18 cm c2c distance (Fig 6D to Fig 6F) appeared to be more dispersed than that for the 36 cm c2c distance (Fig 6A to Fig 6C). As shown in Fig 6E and Fig 6F, estimations with the virtual cursor technique at the narrower (18 cm) c2c distance shifted slightly to the left side of the reference target, while the estimations with the direct selection technique moved towards the right side of the reference target (Fig 6D to Fig 6F). This finding seems to be consistent with a recent study [40] showing that direct pointing estimation accuracy suffers more on the left side of the participants. A plausible explanation for this is the orientation of the interaction technique (direct selection by reaching/pointing to a target), since all the participants were right-handed [40]. The pointing direction of the left-side targets was from the right side of the participant, which would lead to a movement towards the center of the participant’s body. Comparing the corresponding accuracies with those of left-handed participants would provide more information that could possibly explain the possible reasons for the difference. In addition, the fact that the experiment had only two c2c distance levels and that the shifting estimations only occurred in the 18 cm condition may have contributed to the inaccuracy of the user’s perception. A future study should include a greater range of c2c distances to determine whether the present finding of compression of perception in the frontal space can be generalized.

The direct selection technique estimations of x-position (horizontal) and y-position (vertical) of the later projected targets were more accurate than those of the first target (Fig 6). This result may be related to the result from Richardson and Waller [66], who reported that the exocentric distance judgment of participants was improved by the learning effects from training. Since all participants started by pointing at the first target, their judgment could be expected to improve for the later targets.

## Conclusion

In this study, we attempted to compare selection task performance and perception of the frontal extent in a stereoscopic display environment using two interaction techniques: direct selection and a virtual cursor. Selection behavior and performance using direct or indirect interaction techniques have been the concern of several previous works. In addition, this issue is important in areas such as human–machine interaction research. Indeed, virtual reality is now becoming more established and promising, and recent developments have led to applications allowing users to directly perceive and interact with 3D objects rather than just watching a 3D model. The underlying belief of current virtual reality research is that this will lead to more effective human–machine interfaces. In addition, to enrich the knowledge of users’ interactions in virtual environments, comprehensive understanding of users’ interactions in such environments is needed. To develop this understanding, we performed a comparative study with two different interaction techniques by using exactly the same experimental conditions.

In particular, this study was conducted to investigate accuracy in the frontal extent and the corresponding perception of the frontal extent in a stereoscopic environment, where virtual targets of three different sizes were presented at two center-to-center distances and two interaction techniques (direct selection and a virtual cursor) were employed. The results revealed that the accuracies of the x-position, y-position, and exocentric distance in the frontal extent were all affected by the interaction technique. The accuracies were significantly higher for the virtual cursor technique than for the direct selection technique.

Similarly, the results showed that the target size affected the accuracies of the x-position, y-position, and exocentric distance. Accuracy was relatively higher in the large (3.6 cm) target size condition than in the medium (1.5 cm) and small (0.6 cm) target size conditions. There was also an interaction effect between center-to-center distance and technique in the accuracies of the x-position and y-position. In the direct selection condition, it was observed that the accuracies of the x-position and y-position improved as the separation of targets decreased. On the other hand, in the virtual cursor condition, the accuracy of the y-position improved as the separation between targets increased. However, no significant differences in the accuracy of the x-position were found between the two center-to-center distances.

Generally, the accuracy of space perception in the frontal stereoscopic environment was found to be compressed (concentrated to the center) with respect to the target references, as observed from the plots of the pointing estimations. The findings of this study are important for virtual reality application designers and consumers. Developers of manufacturing simulations could potentially improve user accuracy by considering the observed systematic underestimations in the frontal extent. Moreover, the perception of the frontal extent with the direct selection method was found to be underestimated in the wider (36 cm) center-to-center distance condition, although the virtual cursor might have contributed to the reduction of the underestimation. These results thus provide critical information for deciding which interaction techniques can yield better estimation, how virtual targets can be appropriately positioned to enhance users’ performance, and what the relevant target sizes in the frontal extent are so as to reduce judgment errors in stereoscopic environments.

Since accuracy is important in both real-world and virtual environments, the findings of this study imply the necessity of maintaining appropriate levels of both the distance between targets and the size of the virtual targets. Generally, if a direct selection technique is used, the separation between targets should be kept as small as possible, while the target size should be as large as possible. If, on the other hand, a virtual cursor is used, a wider range of size and distance between targets can be accommodated, and the associated accuracy may be relatively higher. Therefore, the virtual cursor technique had an overall relative advantage over the direct selection technique with respect to accuracy under the given experimental conditions.

Furthermore, to assist distance perception as well as possible, it might be important to provide sufficient depth cues by displaying a rich virtual environment containing a texture or gradient background, and by adding objects of familiar sizes to the virtual scene to provide a good sense of presence. The findings of our study should be useful for virtual reality developers in making various decisions, especially in the design of applications such as 3D medical surgery training and manufacturing, where the surgeons and engineers are primarily concerned about the accuracy and precision of interactions.

Finally, it is important to note that this experiment only addressed task performance in a stereoscopic environment projection-based display, using a particular tracking system and input device, and over a particular range of distances between targets (exocentric distance). Understanding the generalizability of the results will require replication of the methodology across a range of virtual environments, displays, and spatial (egocentric or exocentric) configurations.

## References

[1] Chao CJ, Wu SY, Yau YJ, Feng WY, Tseng FY. Effects of three‐dimensional virtual reality and traditional training methods on mental workload and training performance. Human Factors and Ergonomics in Manufacturing & Service Industries. 2017;27(4):187–96. 10.1002/hfm.20702

[2] Webel S, Bockholt U, Engelke T, Gavish N, Olbrich M, Preusche C. An augmented reality training platform for assembly and maintenance skills. Robotics and Autonomous Systems. 2013;61(4):398–403. doi: 10.1016/j.robot.2012.09.013.

[3] de Boer IR, Wesselink PR, Vervoorn JM. Student performance and appreciation using 3D vs. 2D vision in a virtual learning environment. European Journal of Dental Education. 2016;20(3):142–7. 10.1111/eje.12152 26072997

[4] Cutting JE. Reconceiving perceptual space. Looking into pictures: An interdisciplinary approach to pictorial space. Cambridge, MA, US: MIT Press; 2003 p. 215–38.

[5] Kelly JW, Cherep LA, Klesel B, Siegel ZD, George S. Comparison of Two Methods for Improving Distance Perception in Virtual Reality. ACM Trans Appl Percept. 2018;15(2):1–11. 10.1145/3165285

[6] Lin CJ, Woldegiorgis BH, Caesaron D, Cheng L-Y. Distance estimation with mixed real and virtual targets in stereoscopic displays. Displays. 2015;36(Supplement C):41–8. doi: 10.1016/j.displa.2014.11.006.

[7] Lin CJ, Woldegiorgis BH, Caesaron D. Distance estimation of near-field visual objects in stereoscopic displays. Journal of the Society for Information Display. 2014;22(7):370–9. 10.1002/jsid.269

[8] Lin CJ, Woldegiorgis BH. Interaction and visual performance in stereoscopic displays: A review. Journal of the Society for Information Display. 2015;23(7):319–32. 10.1002/jsid.378

[9] Naceri A, Chellali R. Depth perception within peripersonal space using head-mounted display. Presence: Teleoper Virtual Environ. 2011;20(3):254–72. 10.1162/PRES_a_00048

[10] Napieralski PE, Altenhoff BM, Bertrand JW, Long LO, Babu SV, Pagano CC, et al Near-field distance perception in real and virtual environments using both verbal and action responses. ACM Trans Appl Percept. 2011;8(3):1–19. 10.1145/2010325.2010328

[11] Jones JA, Suma EA, Krum DM, Bolas M. Comparability of narrow and wide field-of-view head-mounted displays for medium-field distance judgments. Proceedings of the ACM Symposium on Applied Perception; Los Angeles, California. 2338701: ACM; 2012. p. 119-.

[12] Renner RS, Velichkovsky BM, Helmert JR. The perception of egocentric distances in virtual environments—A review. ACM Comput Surv. 2013;46(2):1–40. 10.1145/2543581.2543590

[13] Cutting JE, Vishton PM. Chapter 3—Perceiving Layout and Knowing Distances: The Integration, Relative Potency, and Contextual Use of Different Information about Depth*. In: Epstein W, Rogers S, editors. Perception of Space and Motion. San Diego: Academic Press; 1995 p. 69–117.

[14] Lappin JS, Shelton AL, Rieser JJ. Environmental context influences visually perceived distance. Perception & Psychophysics. 2006;68(4):571–81. 10.3758/bf03208759 16933422

[15] Witt JK, Stefanucci JK, Riener CR, Proffitt DR. Seeing beyond the target: environmental context affects distance perception. Perception. 2007;36(12):1752–68. Epub 2008/02/21. 10.1068/p5617 . 18283926

[16] Sugovic M, Turk P, Witt JK. Perceived distance and obesity: It's what you weigh, not what you think. Acta psychologica. 2016;165:1–8. Epub 2016/02/09. 10.1016/j.actpsy.2016.01.012 . 26854404

[17] Yamamoto N. Distance Perception In: Kreutzer JS, DeLuca J, Caplan B, editors. Encyclopedia of Clinical Neuropsychology. Cham: Springer International Publishing; 2018 p. 1198–202.

[18] Wartenberg C, Wiborg P. Precision of Exocentric Distance Judgments in Desktop and Cube Presentation. Presence. 2003;12(2):196–206. 10.1162/105474603321640941

[19] Geuss MN, Stefanucci JK, Creem-Regehr SH, Thompson WB. Effect of viewing plane on perceived distances in real and virtual environments. Journal of experimental psychology Human perception and performance. 2012;38(5):1242–53. Epub 2012/03/14. 10.1037/a0027524 . 22409144

[20] Kelly JW, Hammel W, Sjolund LA, Siegel ZD. Frontal extents in virtual environments are not immune to underperception. Attention, Perception, & Psychophysics. 2015;77(6):1848–53. 10.3758/s13414-015-0948-8 26105656

[21] Waller D. Factors Affecting the Perception of Interobject Distances in Virtual Environments. Presence. 1999;8(6):657–70. 10.1162/105474699566549

[22] Dey A, Jarvis G, Sandor C, Reitmayr G, editors. Tablet versus phone: Depth perception in handheld augmented reality. 2012 IEEE International Symposium on Mixed and Augmented Reality (ISMAR); 2012 5–8 Nov. 2012.

[23] Kunz BR, Wouters L, Smith D, Thompson WB, Creem-Regehr SH. Revisiting the effect of quality of graphics on distance judgments in virtual environments: A comparison of verbal reports and blind walking. Attention, Perception, & Psychophysics. 2009;71(6):1284–93. 10.3758/app.71.6.1284 19633344

[24] Loomis JM, Philbeck JW. Measuring spatial perception with spatial updating and action In: Klatzky RL, MacWhinney B, Behrmann M, editors. Embodiment, Ego-Space, and Action. New York: Taylor & Francis, Psychology Press; 2008 p. 1–43.

[25] Swan JE, Livingston MA, Smallman HS, Brown D, Baillot Y, Gabbard JL, et al., editors. A Perceptual Matching Technique for Depth Judgments in Optical, See-Through Augmented Reality. IEEE Virtual Reality Conference (VR 2006); 2006 25–29 March 2006.

[26] Henry D, Furness T, editors. Spatial perception in virtual environments: Evaluating an architectural application. Proceedings of IEEE Virtual Reality Annual International Symposium; 1993 18–22 Sept. 1993.

[27] Altenhoff BM, Napieralski PE, Long LO, Bertrand JW, Pagano CC, Babu SV, et al. Effects of calibration to visual and haptic feedback on near-field depth perception in an immersive virtual environment. Proceedings of the ACM Symposium on Applied Perception; Los Angeles, California. 2338691: ACM; 2012. p. 71–8.

[28] Swan JE, Singh G, Ellis SR. Matching and Reaching Depth Judgments with Real and Augmented Reality Targets. IEEE Transactions on Visualization and Computer Graphics. 2015;21(11):1289–98. 10.1109/TVCG.2015.2459895 26340777

[29] Naceri D, Chellali R, Dionnet F, Toma S. Depth Perception Within Virtual Environments: Comparison Between two Display Technologies. International Journal on Advances in Intelligent Systems. 2010;3:51–64.

[30] Kim K, Rosenthal MZ, Zielinski D, Brady R, editors. Comparison of desktop, head mounted display, and six wall fully immersive systems using a stressful task. 2012 IEEE Virtual Reality Workshops (VRW); 2012 4–8 March 2012.

[31] Alexandrova IV, Teneva PT, Rosa Sdl, Kloos U, B HH, #252, et al. Egocentric distance judgments in a large screen display immersive virtual environment. Proceedings of the 7th Symposium on Applied Perception in Graphics and Visualization; Los Angeles, California. 1836258: ACM; 2010. p. 57–60.

[32] Kelly JW, Cherep LA, Siegel ZD. Perceived Space in the HTC Vive. ACM Trans Appl Percept. 2017;15(1):1–16. 10.1145/3106155

[33] Bruder GS, Fernando Argelaguet; Olivier Anne-Helene; Lecuyer Anatole. CAVE Size Matters: Effects of Screen Distance and Parallax on Distance Estimation in Large Immersive Display Setups. Presence: Teleoperators & Virtual Environments. 2015;25(I):1–16. 10.1109/TCYB.2018.2826016

[34] Grechkin TY, Nguyen TD, Plumert JM, Cremer JF, Kearney JK. How does presentation method and measurement protocol affect distance estimation in real and virtual environments? ACM Transactions on Applied Perception. 2010;7(4):1–18. 10.1145/1823738.1823744

[35] Nguyen A, Banic A, editors. 3DTouch: A wearable 3D input device for 3D applications. 2015 IEEE Virtual Reality (VR); 2015 23–27 March 2015.

[36] Jang Y, Noh S, Chang HJ, Kim T, Woo W. 3D Finger CAPE: Clicking Action and Position Estimation under Self-Occlusions in Egocentric Viewpoint. IEEE Transactions on Visualization and Computer Graphics. 2015;21(4):501–10. 10.1109/TVCG.2015.2391860 26357100

[37] Lubos P, Bruder G, Steinicke F, editors. Analysis of direct selection in head-mounted display environments. 2014 IEEE Symposium on 3D User Interfaces (3DUI); 2014 29–30 March 2014.

[38] Chen J, Or C. Assessing the use of immersive virtual reality, mouse and touchscreen in pointing and dragging-and-dropping tasks among young, middle-aged and older adults. Applied Ergonomics. 2017;65:437–48. 10.1016/j.apergo.2017.03.013 28395855

[39] Lin CJ, Woldegiorgis BH. Egocentric distance perception and performance of direct pointing in stereoscopic displays. Applied Ergonomics. 2017;64(Supplement C):66–74. doi: 10.1016/j.apergo.2017.05.007. 28610816

[40] Woldegiorgis BH, Lin CJ. The accuracy of distance perception in the frontal plane of projection‐based stereoscopic environments. Journal of the Society for Information Display. 2017;25(12):701–11. 10.1002/jsid.618

[41] Bruder G, Steinicke F, Sturzlinger W. To touch or not to touch?: comparing 2D touch and 3D mid-air interaction on stereoscopic tabletop surfaces. Proceedings of the 1st symposium on Spatial user interaction; Los Angeles, California, USA. 2491369: ACM; 2013. p. 9–16.

[42] Jerald J. The VR Book: Human-Centered Design for Virtual Reality: Association for Computing Machinery and Morgan; Claypool; 2016. 635 p.

[43] Mine MR. Virtual Environment Interaction Techniques. University of North Carolina at Chapel Hill, 1995.

[44] Bruder G, Steinicke F, Sturzlinger W. Effects of visual conflicts on 3D selection task performance in stereoscopic display environments. 2013 IEEE Symposium on 3D User Interfaces (3DUI); 16–17 March 20132013. p. 115–8.

[45] Poupyrev I, Ichikawa T. Manipulating Objects in Virtual Worlds: Categorization and Empirical Evaluation of Interaction Techniques. Journal of Visual Languages & Computing. 1999;10(1):19–35. doi: 10.1006/jvlc.1998.0112.

[46] Deng C-L, Geng P, Hu Y-F, Kuai S-G. Beyond Fitts’s Law: A Three-Phase Model Predicts Movement Time to Position an Object in an Immersive 3D Virtual Environment. Human Factors. 2019;61(6):879–94. 10.1177/0018720819831517 . 30912987

[47] Lin CJ, Abreham BT, Woldegiorgis BH. Effects of displays on a direct reaching task: A comparative study of head mounted display and stereoscopic widescreen display. International Journal of Industrial Ergonomics. 2019;72:372–9. doi: 10.1016/j.ergon.2019.06.013.

[48] Poupyrev I, Weghorst S, Fels S. Non-isomorphic 3D rotational techniques. Proceedings of the SIGCHI conference on Human Factors in Computing Systems; The Hague, The Netherlands. 332497: ACM; 2000. p. 540–7.

[49] Werkhoven PJ, Groen J. Manipulation Performance in Interactive Virtual Environments. Human Factors. 1998;40(3):432–42. 10.1518/001872098779591322

[50] Argelaguet F, Andujar C. A survey of 3D object selection techniques for virtual environments. Computers & Graphics. 2013;37(3):121–36. doi: 10.1016/j.cag.2012.12.003.

[51] Chan L-W, Kao H-S, Chen MY, Lee M-S, Hsu J, Hung Y-P. Touching the void: direct-touch interaction for intangible displays. Proceedings of the SIGCHI Conference on Human Factors in Computing Systems; Atlanta, Georgia, USA. 1753725: ACM; 2010. p. 2625–34.

[52] Bruder G, Steinicke F, Stuerzlinger W, editors. Touching the Void Revisited: Analyses of Touch Behavior on and above Tabletop Surfaces 2013; Berlin, Heidelberg: Springer Berlin Heidelberg.

[53] Mine MR, Frederick P. Brooks J, Sequin CH. Moving objects in space: exploiting proprioception in virtual-environment interaction. Proceedings of the 24th annual conference on Computer graphics and interactive techniques. 258747: ACM Press/Addison-Wesley Publishing Co.; 1997. p. 19–26.

[54] Lemmerman DK., LaViola J J. Jr. Effects of Interaction-Display Offset on User Performance in Surround Screen Virtual Environments 2007. 303–4 p.

[55] Wang Y, MacKenzie C. Effects of orientation disparity between haptic and graphic displays of objects in virtual environments. 1999.

[56] Dey A, Cunningham A, Sandor C, editors. Evaluating depth perception of photorealistic mixed reality visualizations for occluded objects in outdoor environments. 2010 IEEE Symposium on 3D User Interfaces (3DUI); 2010 20–21 March 2010.

[57] ISO I. DIS 9241–9: Ergonomic Requirements for Office Work with Visual Display Terminals, Non-Keyboard Input Device Requirements.2000; 1:[57 p.].

[58] Bérard F, Ip J, Benovoy M, El-Shimy D, Blum JR, Cooperstock JR, editors. Did “Minority Report” Get It Wrong? Superiority of the Mouse over 3D Input Devices in a 3D Placement Task 2009; Berlin, Heidelberg: Springer Berlin Heidelberg.

[59] Burno RA, Wu B, Doherty R, Colett H, Elnaggar R. Applying Fitts’ Law to Gesture Based Computer Interactions. Procedia Manufacturing. 2015;3:4342–9. doi: 10.1016/j.promfg.2015.07.429.

[60] Lin J, Harris-Adamson C, Rempel D. The Design of Hand Gestures for Selecting Virtual Objects. International Journal of Human–Computer Interaction. 2019:1–7. 10.1080/10447318.2019.1571783

[61] Shen Y, Ong SK, Nee AYC. Vision-Based Hand Interaction in Augmented Reality Environment. International Journal of Human–Computer Interaction. 2011;27(6):523–44. 10.1080/10447318.2011.555297

[62] Geuss M, Stefanucci J, Creem-Regehr S, Thompson WB. Can I pass?: using affordances to measure perceived size in virtual environments. Proceedings of the 7th Symposium on Applied Perception in Graphics and Visualization; Los Angeles, California. 1836259: ACM; 2010. p. 61–4.

[63] Interrante V, Ries B, Anderson L, editors. Distance Perception in Immersive Virtual Environments, Revisited. IEEE Virtual Reality Conference (VR 2006); 2006 25–29 March 2006.

[64] Interrante V, Ries B, Lindquist J, Kaeding M, Anderson L. Elucidating Factors that Can Facilitate Veridical Spatial Perception in Immersive Virtual Environments. Presence: Teleoperators and Virtual Environments. 2008;17(2):176–98. 10.1162/pres.17.2.176

[65] Sun H-M, Li S-P, Zhu Y-Q, Hsiao B. The ef\fect of user's perceived presence and promotion focus on usability for interacting in virtual environments. Applied Ergonomics. 2015;50:126–32. 10.1016/j.apergo.2015.03.006 25959326

[66] Richardson AR, Waller D. The effect of feedback training on distance estimation in virtual environments. Applied Cognitive Psychology. 2005;19(8):1089–108. 10.1002/acp.1140

